# Our Terms, Cash

**Published:** 1873-05

**Authors:** 


### PART III.

### EDITORIAL AND MISCELLANEOUS.

*Our Terms, Cash.*

The subscription price of the RECORD is low—very, when the amount and variety of reading matter given during the year is considered. In keeping the price low, we have been compelled to assume the risk of making it a success, and therefore cannot afford to fail by running it on the credit system. Two and a half dollars is a very small sum to the reader, but to the publisher the aggregate is by no means inconsiderable. Please remember that it requires so much cash a month to lay the RECORD before its readers, and we hope all friends, while pardoning these remarks, will at once send on the amounts of their subscriptions. Don't delay a single day after reading this.

